# 

**Published:** 1906-07

**Authors:** 


### CONTENTS.

ORIGINAL COMMUNICATIONS—

Another Case of Priapism from Nasal Reflex. By Arthur G. Hobbs, M.D., Atlanta, Ga ..... 217

Diagnosis and Treatment of Pulmonary Tuberculosis. By H. F. Harris, M.D., Atlanta, Ga ..... 223

Radium. By F. G. Hodson, M.D., Atlanta, Ga ..... 228

Malaria—Its Treatment. By Charles Hayes, M.D., Vinemont, Ala ..... 240

Recent Studies in Rabies. By James N. Brawner, M.D., Atlanta, Ga ..... 245

The Giant Magnet as an Eye Preserver. By Ross P. Cox, Rome, Ga ..... 251

Friedreich's Disease. By Wesley E. Taylor, M.D ..... 254

EDITORIAL NOTES AND COMMENTS—

Cigarettes and the Boys ..... 257

Not to be Aligned ..... 260

NEWS AND NOTES ..... 262

CONTENTS CONTINUED ..... x

